# Lactate values during labour and their correlation with maternal and fetal outcome

**DOI:** 10.1530/RAF-25-0140

**Published:** 2026-03-24

**Authors:** Barbara Milan, Giulia Fierro, Ilaria Roncagliolo, Francesca Graziano, Vicky Rubini, Nilaya Signori, Elena Buelli, Santa Maria Barresi, Ferdinando L Lorini

**Affiliations:** ^1^ Department of Emergency and Intensive Care, ASST Papa Giovanni XXIII, Bergamo, Italy; ^2^ Department of Anaesthesia and Perioperative Medicine, The Royal London Hospital, London, UK; ^3^ Biostatistics and Clinical Epidemiology, Fondazione IRCCS San Gerardo Dei Tintori, Monza, Italy; ^4^ University of Milan, Milan, Italy; ^5^ Department of Obstetrics and Gynaecology, ASST Bergamo EST, Bergamo, Italy; ^6^ Department of Obstetrics and Gynaecology, ASST Papa Giovanni XXIII, Bergamo, Italy

**Keywords:** lactate, labour physiology, uterine contractions, hypoxia-induced force increase, postpartum haemorrhage, maternal metabolism

## Abstract

**Graphical Abstract:**

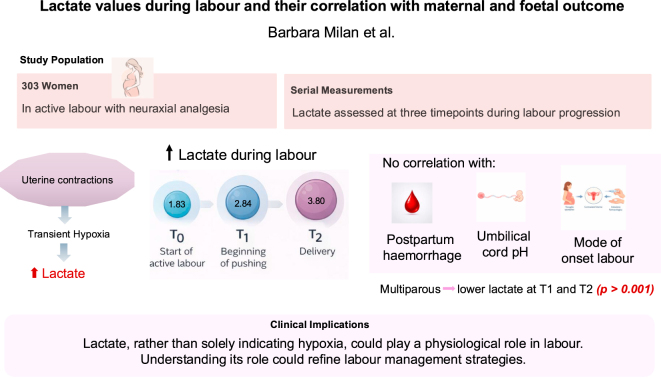

**Abstract:**

This study investigates the relationship between lactate levels – produced during myometrial contraction and transient hypoxia – and maternal and fetal outcomes. Uterine contractions during labour are regulated by hormonal and metabolic processes, including oxytocin release, catecholamine modulation, and the hypoxia-induced force increase (HIFI) mechanism. In a cohort of 303 women receiving neuraxial analgesia, lactate was measured at three time points: at the diagnosis of the active phase of the first stage of labour (T0), at the beginning of active pushing (T1), and at delivery (T2). Lactate values increased progressively from T0 to T2, with median values exceeding 2 mmol/L at T1 and T2 (T1 = 2.84, SD = 1.32, and T2 = 3.80, SD = 1.56). However, no significant correlation was found between lactate levels and postpartum haemorrhage; both blood loss and the interaction term (time and blood loss) were not statistically significant (*P* = 0.574 and *P* = 0.211, respectively). Lactate levels did not significantly correlate with the mode of onset of labour or neonatal umbilical cord pH alterations. Instead, multiparous women exhibited lower lactate levels at T1 and T2 (*P* < 0.001) compared with nulliparous women, likely due to a more efficient myometrial response and metabolic adaptation. These findings suggest that lactate, rather than solely indicating hypoxia, could play a physiological role in labour and should not be regarded exclusively as a marker of distress. Understanding its role could refine the clinical interpretation of maternal metabolic stress and improve labour management strategies.

**Lay summary:**

Many women die after childbirth because of severe bleeding. Bleeding occurs mostly when the uterus is unable to contract properly. Identifying a blood biomarker that defines this risk during labour would be important. We considered lactate a possible biomarker. Lactate is produced by cells when oxygen levels drop, as may happen during labour when the uterus contracts rhythmically for many hours. We measured lactate levels in 303 women who had a type of pain relief known as neuraxial analgesia, where a local anaesthetic is given into the spine. We found that lactate levels increased during labour and differed depending on whether a woman had given birth before or not. However, lactate levels were not linked to postpartum haemorrhage, nor did they affect the baby’s well-being. Our findings suggest that lactate is not always a sign of distress but rather a potential normal factor to enhance the understanding and management of labour.

## Introduction

Labour represents the physiological process whereby involuntary, rhythmic, and peristaltic contractions of the uterus lead to the effacement and dilation of the cervix, allowing the baby to pass through the birth canal. Rhythmic uterine contractions are controlled by several factors, including the release of catecholamines, oxytocin, and prostaglandins and a complex process called hypoxic-induced force increase (HIFI) (Supplementary Fig. 3 (see section on Supplementary materials given at the end of the article)) (Arrowsmith & Wray 2014, Arrowsmith *et al.* 2014, Alotaibi *et al.* 2015, Buckley 2015, Wray *et al.* 2021).

Endogenous catecholamine concentrations increase throughout labour due to stress, pain, and physical effort, modulating uterine smooth muscle contractions, increasing endorphin production, and promoting the release of oxytocin (Buckley 2015). Adrenaline increases eightfold during labour compared to baseline levels and has a tocolytic effect, while noradrenaline has a uterotonic action (Buckley 2015). Although both adrenaline and noradrenaline are increased, a tocolytic effect predominates; this effect is antagonised by oxytocin (Segal *et al.* 1998). Furthermore, oxytocin stimulates the production of prostaglandins, which enhances the myometrium’s sensitivity to oxytocin (Buckley 2015).

The last crucial process that regulates uterine contractility is the mechanism of HIFI. This mechanism was studied *in vivo* in pregnant rats; currently, it is very difficult to study this phenomenon in pregnant women (Wray *et al.* 2021). HIFI occurs in the presence of oxytocin, but even when all oxytocin receptors are saturated, HIFI promotes an increase in the myometrium’s contractile strength (Alotaibi *et al.* 2015).

The myometrium contraction occludes uterine blood vessels and induces a brief hypoxic state, resulting in elevated lactate levels, a decreased pH, and a reduced intracellular concentration of adenosine triphosphate (ATP), which promotes the subsequent relaxation of smooth muscle fibroblasts (Supplementary Fig. 3 Db) (Alotaibi *et al.* 2015).

Hypoxia inhibits voltage-operated calcium channels (VOCCs) and decreases myometrial contractility (Wray *et al.* 2021). However, the efflux of ATP from the myometrial cells preserves intracellular calcium concentration, and the hydrolysis of ATP increases myosin light chain kinase (MLCK) activity while decreasing myosin light chain phosphatase (MLCP) activity. It has been hypothesised that the hypoxic phase of HIFI influences calcium sensitivity rather than increasing intracellular calcium entry, and the inhibition of MLCP increases the strength of subsequent uterine contractions (Alotaibi *et al.* 2015). This complex mechanism occurs only in the term-pregnant myometrium, with no analogous response (regarding enhanced contractile strength) shown in the preterm or non-pregnant myometrium (Alotaibi *et al.* 2015).

The standard lactate threshold in healthy people is up to 2 mmol/L (Marik & Bellomo 2013). During labour, lactate levels higher than 2 mmol/L are observed (Nordström *et al.* 2001, Bauer *et al.* 2019). Currently, reference ranges for lactic acid levels during labour remain undefined.

Lactic acid is hypothesised to play a role in the mechanisms regulating labour. There is evidence of its function as an inflammation inhibitor (Madaan *et al.* 2017); also, it has been shown that high lactate levels and a low pH exert a tocolytic effect on the myometrium (Hanley *et al.* 2015). Hanley *et al.* (2015) demonstrated *in vitro*, in pregnant rat myometrium, that lactate and other weak acids cause a dose-dependent decrease in contractility, even if the presence of oxytocin reduces this tocolytic effect.

As lactate may affect the tone of the uterus, elevated levels during labour could influence maternal and fetal well-being. We decided to assess lactate concentrations during labour in women receiving neuraxial analgesia and evaluate the association between lactate levels and adverse maternal and fetal outcomes. The primary aim of our study was to assess whether lactate values during labour were associated with an increased risk of postpartum haemorrhage (PPH). Postpartum haemorrhage represents a significant cause of morbidity in the obstetric population, with a global mortality rate of 140,000 women per year (Graham *et al.* 2016). The predominant cause of PPH (70%) is uterine atony (Solomon *et al.* 2012). Our secondary aims were to assess lactate levels at different stages of labour and investigate whether there was any association between lactate concentrations and neonatal cord pH on delivery.

## Materials and methods

### Study design and participants

We conducted a prospective observational single-centre study involving women in active labour receiving neuraxial analgesia. The study design was approved by the Ethical Committee of ASST Papa Giovanni XXIII Hospital in May 2023 (REF: no. 751/2023, reg 56/23) (ClinicalTrials.gov identifier NCT06807255). The study was designed and presented in accordance with the STROBE guidelines (von Elm *et al.* 2014).

Consecutive women in labour with neuraxial analgesia were recruited from May to September 2023 in the labour ward of ASST Papa Giovanni XXIII Hospital (Bergamo, Italy), a tertiary care referral centre (Italian Ministerial Decree no. 70/2015) for obstetric care in the north of Italy. Before enrolment, all women received written information about the study and the processing of their personal and clinical data, and they signed an informed consent form.

Patients included in the study were ≥18 years old, had a single pregnancy with a gestational age of ≥36 weeks, were in active labour with a cervix dilation of 4–6 cm, received neuraxial analgesia, and gave their consent to the study.

Exclusion criteria were age < 18 years, twin pregnancy, gestational age < 36 weeks, women in labour without labour analgesia, cervix dilation >6 cm, and women who did not consent to the study. Patients who did not undergo vaginal delivery were excluded from the study.

### Study outcomes

The primary aim of our study was to assess whether lactate levels at three specific time points are associated with postpartum haemorrhage in different types of labour. The secondary aims were to assess lactate levels at different stages of labour, compare them depending on parity and mode of labour onset, and assess whether there is any association between lactate concentrations and neonatal pH at delivery.

### Procedures – labour analgesia

Labour analgesia was placed at the beginning of active labour, which is considered to be between 4 and 6 cm of cervical dilatation (ACOG 2019, WHO 2020, NICE 2022). The labour analgesia technique consisted of standard epidural or dural puncture epidural (DPE); analgesia was maintained by intermittent clinician boluses on maternal request. The chosen local anaesthetic was ropivacaine, whose concentration was increased (from 0.1% up to 0.15%, for a total volume of 15–20 mL) based on cervical dilatation and fetal head position at the time of the bolus administration. For each bolus, sufentanil was added (10–5 μg in epidural technique and 7.5–3.75 μg in DPE).

### Data collection

We recorded the following demographic and clinical information: age, pre-pregnancy and post-pregnancy body mass index (BMI), gestational age, parity, mode of onset of labour: spontaneous vs induction, different methods of labour induction, duration of each stage of labour, total postpartum blood loss at the end of the third stage, and umbilical artery fetal pH.

Lactate levels were assessed by blood gas analysis on samples obtained from the women’s indwelling peripheral venous cannulae; subsequent analysis was conducted using the point-of-care emogasanalyser 500 systems (Siemens, Milano) at three time points. The three time points were as follows: T0: at the time of diagnosis of the active phase of the first stage of labour, T1: at the beginning of the active phase of the second stage of labour, at the beginning of active pushing, and T2: at the time of the delivery, before placenta expulsion.

Postpartum haemorrhage has been defined following the definition of the Royal College of Obstetricians and Gynaecologists as a total blood loss ≥500 mL (BJOG 2017). Our population was divided into two groups: those with total blood loss <500 mL and those with blood loss ≥500 mL.

To measure umbilical cord pH, 1–2 mL of cord blood was taken from the umbilical artery one minute after birth, regardless of clamping. The analysis was conducted with the point-of-care emogasanalyser 500 systems blood gas analyser (Siemens, Milan).

### Statistical analysis

Baseline characteristics were described as mean (standard deviation (SD)), median (I–III quartiles), and absolute and relative frequencies, as appropriate. Differences between groups (e.g. nulliparous/multiparous) of lactate levels at each time point were assessed by *t*-tests or Mann–Whitney U tests, following the nature of the variable. A preliminary analysis to explore the relationship between postpartum blood loss as a continuous variable and maternal lactate levels was performed using the Spearman’s rank correlation (*r*) and its associated *P*-value. To evaluate the variation of lactate over time, an initial exploratory analysis was conducted graphically using a spaghetti plot. Mixed-effects models were applied to evaluate the effect of postpartum haemorrhage, modelled both dichotomously and continuously, and other clinically relevant variables on lactate variation. Finally, the relationships between lactate levels at each time point and the neonatal pH (expressed in logarithmic scale) were also explored by the Spearman’s rank correlation (*r*) and its associated *P*-value. The tests were two-sided with a type I error set at a significance level of 0.05. All analyses were performed using R (version 4.3.1).

## Results

### Study population

Between May and September 2023, we enrolled 381 women who were in active labour and had received neuraxial analgesia. Thirty-one cases were excluded from the analysis due to caesarean delivery, and forty-seven were excluded due to incomplete data (the enrolment process is shown in Supplementary Fig. 1). We conducted the final analysis on 303 women, 242 of whom were nulliparous and 61 were multiparous.

The demographic, clinical, and labour characteristics of our study population are shown in Table 1. The mean age was 32.02 (SD = 4.71) years, with a mean gestational age of 39.59 weeks, and the median pre-pregnancy BMI was equal to 21.8 (I–III quartiles = 19.8–24.2).

**Table 1:** Demographic and clinical data of the study population. Data are presented as *n* (%), median (I–III quartiles), or mean ± SD.

Characteristics	Values
*n*	303
Age, years	32.02 ± 4.71
BMI	
Pre-pregnancy	21.80 (19.80, 24.20)
Post-pregnancy	26.60 (24.65, 29.45)
Gestational age ≥ 40 weeks	138 (45.5%)
Gestational age, weeks	39.59 ± 1.19
Nulliparous	242 (79.9%)
Type of labour	
Spontaneous labour	134 (44.2%)
Spontaneous labour + augmentation with oxytocin	57 (18.8%)
Induced labour	
Induction with mechanical methods*	13 (4.3%)
Induction with prostaglandins^†^	35 (11.6%)
Induction with oxytocin only	17 (5.6%)
Induction by a combination of methods^‡^	47 (15.5%)
Length of stage, minutes	
First stage	240.00 (137.50, 315.00)
Second stage	47.00 (25.50, 75.00)

BMI, body mass index.

* Double-balloon catheters or amniotomy.

^†^ E2 vaginal or E1 oral.

^‡^ Induction with prostaglandins or mechanical methods and amniotomy and oxytocin.

### Lactate levels over time

**Figure 1:**
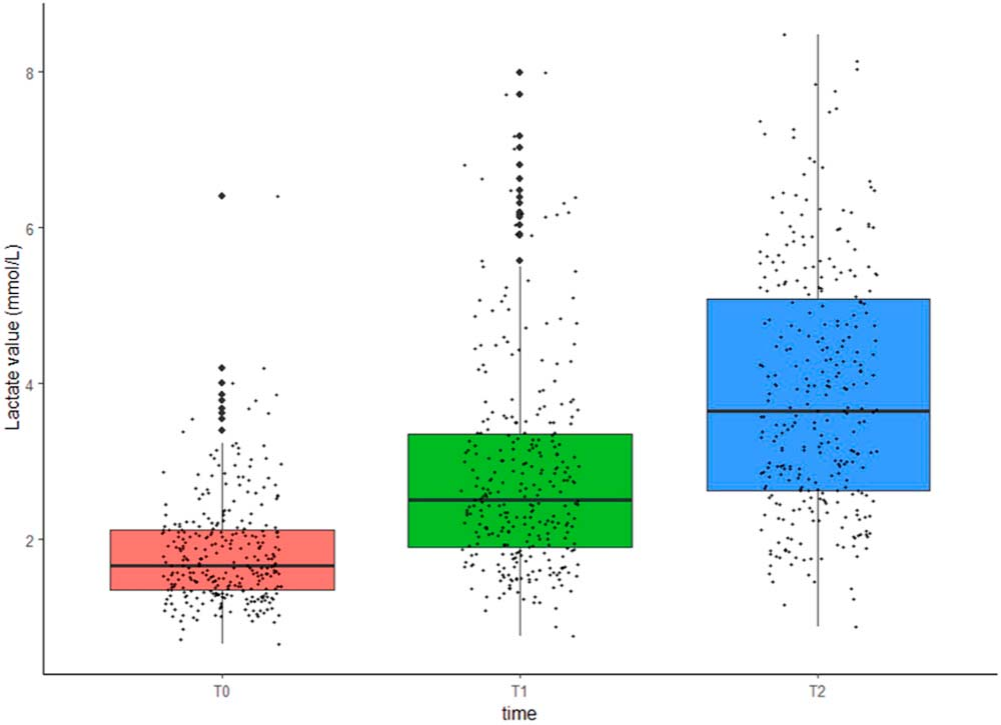
Distribution of lactate levels at each time point measured.

Figure 1 illustrates the distribution of lactate levels at each time point measured. The mean at T0 was 1.83 (SD = 0.68), and it increased over time from the onset of labour to delivery. At T1 and T2, median lactate values are higher than 2 mmol/L (T1 = 2.84, SD = 1.32, and T2 = 3.80, SD = 1.56).

### Postpartum haemorrhage

The median total postpartum blood loss was 400 mL (I–III quartiles = 300–600 mL, min–max = 100–2,400 mL). A total of 110 (36.3%) patients experienced blood loss of more than 500 mL; a total of 34 (11.2%) had a blood loss over 1,000 mL, and 8 patients (2.6%) had a blood loss over 1,500 mL.

**Figure 2:**
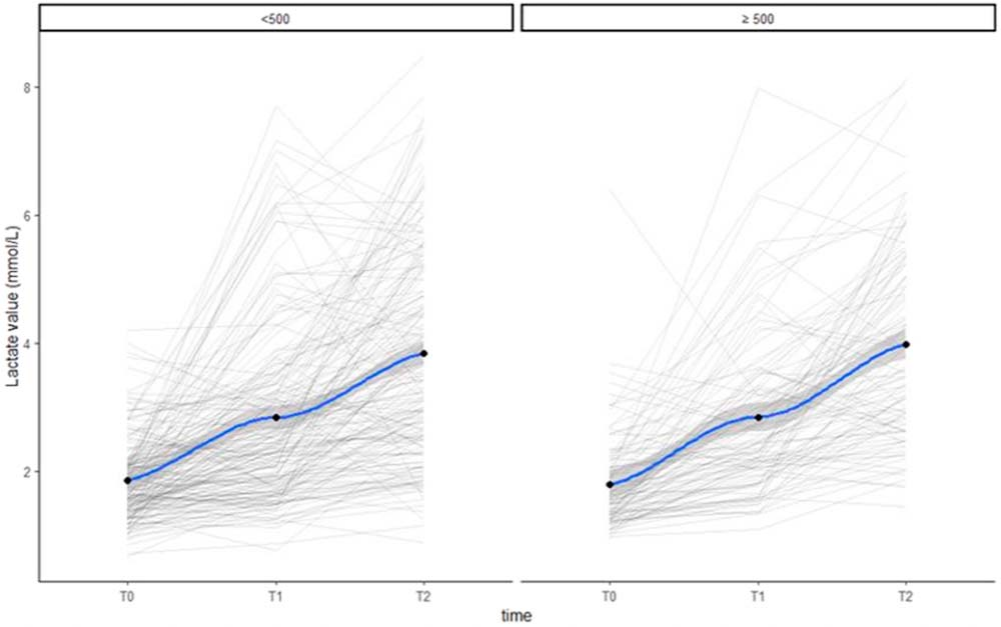
Lactate values at the three time points and changes over time in the blood loss groups.

As shown in Supplementary Fig. 2, the correlation between the blood loss and lactate values at each time point was not statistically significant. No differences were also observed between the two blood loss groups (≥500 mL vs <500 mL) in lactate values at the three time points and in changes over time from T0 (Table 2A and B; Fig. 2). Considering the linear mixed models, both blood loss, analysed as a categorical (≥500 vs <500 mL) and as a continuous variable, and the interaction term between time and blood loss were not statistically significant (*P* = 0.574 and *P* = 0.211 using the 500 mL cut-off; *P* = 0.908 and *P* = 0.624 when blood loss was modelled as a continuous variable). Further analysis showed no statistically significant difference in lactate values even with blood losses >1,000 mL (*n* = 26) (*P* = 0.446) or >1,500 mL (*n* = 8) (*P* = 0.471).

**Table 2:** Lactate levels (mean ± SD) at each time point (A) and changes from baseline (T0) over time (B) in women with postpartum blood loss ≥500 and <500 mL.

	Blood loss		Mean difference* (95% CI)	*P*
	<500 mL	≥500 mL		
A. Time point				
T0	1.85 ± 0.65	1.78 ± 0.75	0.07 (−0.09; 0.24)	0.406
T1	2.83 ± 1.38	2.84 ± 1.22	0.01 (−0.31; 0.29)	0.947
T2	3.84 ± 1.61	3.98 ± 1.49	0.14 (−0.50; 0.22)	0.439
B. Change from baseline				
T0–T1	0.98 ± 1.35	1.058 ± 1.26	0.07 (−0.39; 0.22)	0.601
T0–T2	1.99 ± 1.51	2.19 ± 1.42	0.21 (−0.56; 0.13)	0.223

* Difference in blood loss: <500 mL – blood loss >500 mL.

**Figure 3:**
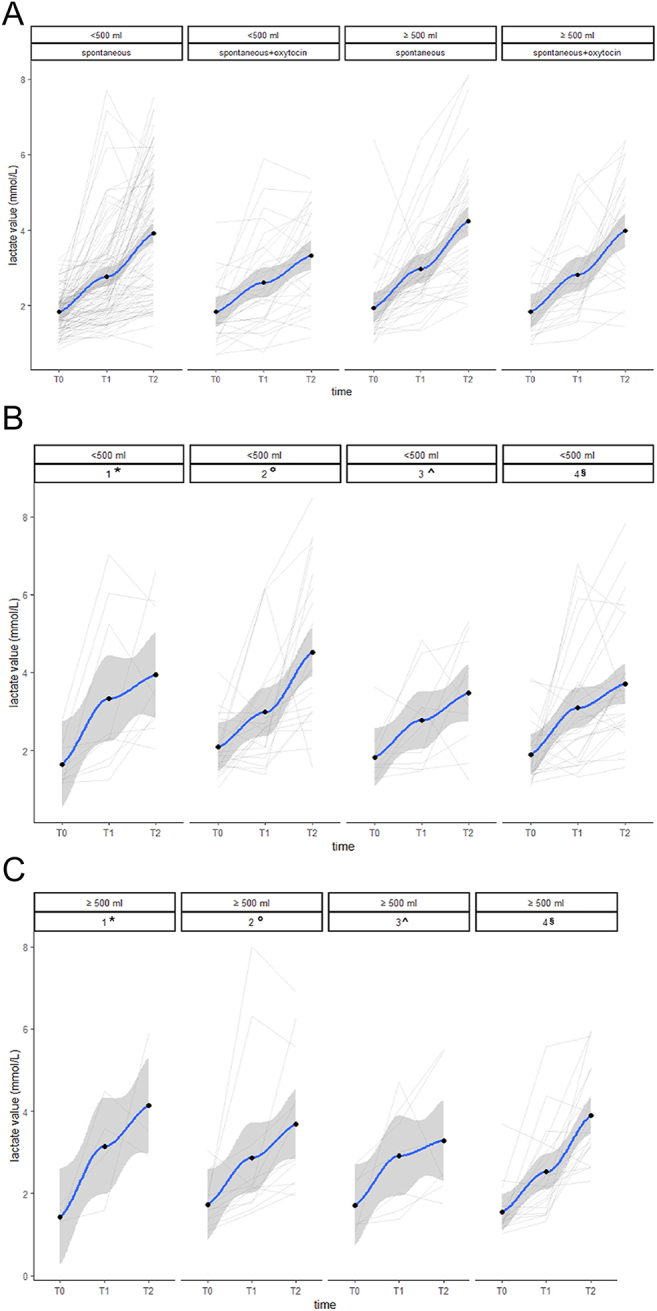
Comparison of groups based on mode of labour. 1*: Induction with only mechanical methods (double-balloon catheters or amniotomy); 2°: induction with only prostaglandins (E2 vaginal or E1 oral); 3^: induction with only oxytocin; 4§:  induction with  prostaglandins or mechanical methods and amniotomy and oxytocin.

An increase in lactate value over time was shown in all types of labour (Supplementary Table 1). No statistical differences were found in the trend over time of lactate values and blood losses in the different types of labour. No statistical differences were found comparing spontaneous labour vs spontaneous labour with oxytocin augmentation (Fig. 3, *P* > 0.8), and no differences were found comparing different methods of induction (induction with only mechanical methods vs induction with only prostaglandins vs induction with only oxytocin vs induction with prostaglandins or mechanical methods and amniotomy and oxytocin, Fig. 3, *P* > 0.9). Lactate values at the three time points in different groups of induction or spontaneous labour are reported in Supplementary Tables 2 and 3.

**Figure 4:**
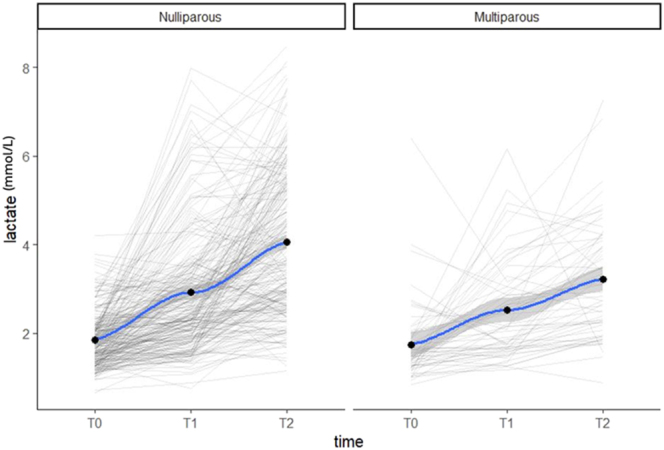
Comparison of groups based on parity.

### Lactate, parity, and labour induction

Of the 303 patients, 242 were nulliparous and 61 were multiparous. Detailed results are illustrated in Table 3.

**Table 3:** Lactate values at the three time points and change in lactate values over time among the two groups (nulliparous and multiparous). Data are expressed as mean ± SD. Statistically significant *P* values are in bold.

	Nulliparous (*n* = 242)	Multiparous (*n* = 61)	Mean difference* (95% CI)	*P*
Time				
T0	1.85 ± 0.62	1.75 ± 0.88	0.10 (−0.13; 0.35)	0.3721
T1	2.92 ± 1.36	2.53 ± 1.10	0.39 (0.06; 0.72)	**0.019**
T2	4.06 ± 1.58	3.23 ± 1.30	0.83 (0.45; 1.22)	**<0.001**
Changes in time				
T0–T1	1.06 ± 1.30	0.77 ± 1.35	0.29 (−0.09; 0.67)	0.141
T0–T2	2.21 ± 1.45	1.47 ± 1.45	0.80 (0.32; 1.14)	**<0.001**

* Difference between nulliparous and multiparous.

As shown, significant differences were found among nulliparous and multiparous women at time points T1 and T2 (differences among groups: 0.39, 95% CI = 0.06, 0.72, *P* = 0.019, and 0.83, 95% CI = 0.45, 1.22, *P* < 0.001, at T1 and T2, respectively). This result is confirmed considering the lactate change in time T2 – T0 (0.80, 95% CI = 0.32, 1.14, *P* < 0.001; Table 3 and Fig. 4) and in the linear mixed model (interaction term, *P* < 0.001).

Regarding the mode of onset of labour, 191 women entered labour spontaneously, while 112 women underwent an induction of labour.

Regarding the mode of onset of labour, 191 women entered labour spontaneously, while 112 women underwent an induction of labour. In these two groups, the lactate trend did not show statistically significant differences at T0, T1, and T2, as well as in the change from T0 (Table 4).

**Table 4:** Lactate values at the three time points and change in lactate values over time among the two groups and mode of onset of labour. Data are presented as mean ± SD.

	Induced labour (*n* = 112)	Spontaneous labour (*n* = 191)	Mean difference* (95% CI)	*P*
Time				
T0	1.80 ± (0.67)	1.85 ± (0.69)	0.05 (−0.11; 0.21)	0.511
T1	2.93 ± (1.49)	2.79 ± (1.22)	0.14 (−0.47; 0.19)	0.404
T2	3.89 ± (1.59)	3.90 ± (1.55)	0.01 (−0.36; 0.38)	0.959
Changes in time				
T0–T1	0.94 ± (1.18)	1.13 ± (1.51)	0.19 (−0.52; 0.14)	0.251
T0–T2	2.04 ± (1.43)	2.08 ± (1.55)	0.04 (−0.39; 0.31)	0.808

* Difference between induced and spontaneous labour.

### Lactate and fetal pH

**Figure 5:**
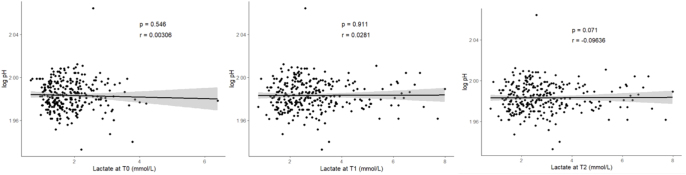
Correlation between lactate at the three distinct time points and fetal pH.

The median value of fetal pH (umbilical artery sampling) was 7.26, I–III quartiles = 7.2–7.3. There was no statistically significant correlation between lactate levels at any time point and fetal pH: at T0, the correlation index *r* = 0.03, *P* = 0.546; at T1, *r* = 0.03, *P* = 0.911; and at T2, *r* = −0.1, *P* = 0.071 (Fig. 5).

## Discussion

It has been hypothesised that lactic acid acts as an inhibitor of inflammation and that low pH and high lactate levels may have a tocolytic effect on the myometrium (Madaan *et al.* 2017). Our study shows no statistically significant difference in lactate values at the three time points, between women with blood losses less than or equal to and more than 500 mL (Table 2). Subsequent exploratory analysis confirmed that this finding remained consistent for blood losses exceeding 1,000 or 1,500 mL. No statistically significant association was observed even when blood loss was analysed as a continuous variable.

Labour is a complex and strenuous experience that leads to a hypermetabolic state and substantial energy production. Such production is predominantly facilitated by adrenaline, which induces glycogenolysis and enhances glucose availability. The myometrium of pregnant humans appears to use glucose as its only energy source (Wray *et al.* 2021). Hypoxic events inhibit the oxidative pathway of the Krebs cycle and excessive glycolysis produces elevated pyruvate levels exceeding mitochondrial oxidative capacity, thus promoting lactic acid production (Marik & Bellomo 2013). During labour, lactate levels appear to increase in association with recurrent hypoxic episodes during myometrial contractions and increased glycogenolytic activity, potentially exceeding the oxidative capacity of pyruvate metabolism.

The possible reason why lactate levels and postpartum haemorrhage were not statistically significant is that the increased lactate during labour should probably not be regarded exclusively as a biomarker of hypoxia and cellular damage but as a typical physiological response of the organism. Furthermore, the tocolytic effect of lactate is likely reduced by endogenous oxytocin, as demonstrated by *in vitro* studies (Hanley *et al.* 2015).

We even analysed lactate values, classifying patients based on the labour type: spontaneous, spontaneous with oxytocin augmentation, or induced labour. Labour induction and oxytocin augmentation are risk factors for PPH (Sheldon *et al.* 2014, Bunch *et al.* 2018).

In our study, the lactate value increases over time in all types of labour, but no statistical differences were found in the trend over time of lactate and blood losses in the different types of labour (Fig. 3). Therefore, lactate values do not correlate with an increased incidence of PPH, even in induced or augmented labour, supporting the hypothesis that lactate values are likely a typical physiological response of the organism during labour.

In the general population, plasma lactate levels remain up to 2 mmol/L in health (Marik & Bellomo 2013).

In our population, at the onset of active labour (T0), the average lactate values were below 2 mmol/L, while at the start of the active phase of the second stage (T1) and at delivery (T2), the median lactate values were higher than 2 mmol/L (Fig. 1). This is in keeping with a meta-analysis by Baur *et al.*  (Bauer *et al.* 2019) that assessed lactate concentrations in healthy pregnant women during gestation and labour. They identified 22 studies involving 1,193 patients to show that women have venous lactate levels below 2 mmol/L during pregnancy and between 2 and 4 mmol/L during active labour. During the second stage and at delivery, some studies reported lactate levels above 4 mmol/L. The limitation of this meta-analysis is that it includes studies whose populations analysed show significant heterogeneity.

Labour is a complex phenomenon influenced by numerous elements, including parity and the mode of onset of labour (Spandrio 2014, NICE 2022). Our sub-analysis indicates that lactate generation during labour is diminished in multiparous women compared to nulliparous women, with statistically significant differences observed at T1 and T2 (*P* = 0.019 and *P* < 0.001, respectively; see Table 3). Our findings are echoed by the research conducted by Schneider *et al.* (1990). However, the population analysed in that study did not receive neuraxial analgesia (Schneider *et al.* 1990).

Multiparous women likely exhibit reduced metabolic exertion during the expulsion phase, which is typically more rapid and has more favourable outcomes, with a diminished risk of surgical intervention compared to nulliparous women (Spandrio 2014, Ashwal *et al.* 2020). Parity may affect activation levels of myometrial cells during labour; specifically, in multiparous women, the receptors for oxytocin are more sensitive and easily inducible (Coad 2019).

Finally, various hormonal mechanisms are implicated, including a difference in beta-endorphin concentration between multiparous and nulliparous women (Weissberg *et al.* 1990, Buckley 2015). All these characteristics likely lead to a less significant increase in lactate at the end of the second stage. This supports the role of lactic acid as a physiological marker of metabolic stress of labour. The literature reports that induced labour is more painful and prolonged (Spandrio 2014, Jacobsen *et al.* 2018, Chestnut 2019). However, our study did not observe any statistically significant differences in lactate levels between induced and non-induced labour (Table 4). To our knowledge, no previous study has compared lactate production in relation to the mode of labour onset.

As all participants in our study received analgesia, we cannot exclude the possibility that neuraxial analgesia influenced our findings. The role of neuraxial analgesia in modulating the neuroendocrine aspects of labour is well established. By modulating pain, neuraxial analgesia diminishes catecholamine levels during labour (Buckley 2015, Andrade Santos *et al.* 2022), reduces endorphin production, and decreases oxytocin concentrations (Buckley 2015). These neuroendocrine effects may have attenuated potential differences in lactate production between spontaneous and induced labour. These modifications highlight the challenges of comparing women with analgesia with women without analgesia during labour (Buckley 2015).

As early as 1991, Piquard *et al.* was among the first to recognise the significance of differentiating between women undergoing neuraxial analgesia and those not receiving analgesia (Piquard *et al.* 1991). Comparing our findings with Piquard’s, lactate levels at T2 in our population are almost 1 mmol higher than levels reported by Piquard in patients with neuraxial analgesia. This discrepancy may be attributed to the prevalence of multiparous patients compared to primiparous patients in the cohort examined by Piquard, who did not perform a sub-analysis in nulliparous and multiparous women.

Intrapartum fetal hypoxia potentially increases the fetal risk of asphyxia, acidosis, neuronal damage, long-term morbidity, or even death (Allanson *et al.* 2017). Blood gas analysis of umbilical blood samples is recognised in the literature as the only method for objectively measuring the incidence of fetal acidosis or hypoxia (Ayres-de-Campos & Arulkumaran 2015).

Among the secondary endpoints, we studied the correlation between maternal venous lactate and fetal pH.We have shown no statistically significant correlation between lactate at the three distinct time points and fetal pH (Fig. 5).

Our findings corroborate those previously published by Piquard *et al.* (1991). The authors observed that the lactate concentration in the mother’s venous blood and cord arterial blood was in equilibrium at birth after elective caesarean sections. However, during labour, fetal and maternal lactate values changed independently. The authors discovered that in hyporeactive newborns, the lactate concentration in cord blood exceeded that in maternal venous blood, suggesting fetal lactate production (Piquard *et al.* 1991).

The absence, in our study, of significant correlation between maternal lactate and fetal pH is consistent with previous evidence and suggests that these parameters reflect distinct aspects of metabolic and acid–base change occurring during labour. Our findings indicate that maternal lactate levels should be interpreted independently from fetal pH, although further analyses are required to study the relationship between maternal lactate values and fetal well-being. Overall, these findings should be interpreted considering the study’s strengths and limitations. The study population is homogeneous because they all had neuraxial analgesia during labour.

An increasing number of women are requesting neuraxial analgesia during labour to modulate pain. In the USA, approximately 68.9% (Parameshwar *et al.* 2024) of patients in labour receive analgesia, while in our hospital, the percentage is around 50%. Considering the widespread use of this technique, it is important to study how lactate levels vary during these labours.

Regarding other potentially influential variables, these were identified (parity and induction) and analysed, with the study cohort being stratified to avoid significant confounding factors as far as possible. While this enhances data reliability, it remains a daunting challenge to consider all the variables present in the obstetric population and in labour.

Our study has a major limitation. Our findings are applicable only to women with neuraxial analgesia. In our hospital, anaesthetists oversee labour in women receiving neuraxial analgesia. Unmedicated labours are managed by midwives, and the anaesthetist is involved only in case of complications. Therefore, for us, it is very difficult to enrol women who do not undergo neuraxial analgesia. Further research is necessary to investigate lactate values in women without analgesia.

## Conclusion

Labour is a complex physiological event with specific metabolic characteristics. Elevated levels of lactate during labour may represent a physiological source of energy, produced and utilised by the uterus under stressful conditions, and not exclusively a marker of cellular hypoxia. This is demonstrated by the absence of a correlation between maternal lactate and adverse maternal–fetal outcomes. Clinicians could benefit from understanding the ‘physiological’ lactate values during and after delivery to evaluate the progress of labour and to ensure the accurate interpretation of lactacidaemia, even in the event of complications in the immediate postpartum period. Further research is essential to clarify the role of lactate during labour.

## Supplementary materials



## Declaration of interest

The authors declare that there is no conflict of interest that could be perceived as prejudicing the impartiality of the work reported.

## Funding

This research did not receive any specific grants from funding agencies in the public, commercial, or not-for-profit sectors.

## Author contribution statement

BM and GF conceptualised the study and drafted the manuscript. IR drafted the manuscript and acquired the data. FG analysed and interpreted the data. VR drafted the manuscript. NS, EB, and SMB acquired the data. FLL supervised the study. All authors read and approved the final version of the manuscript for publication.

## Data availability

The datasets generated during and/or analysed during the current study are available from the corresponding author on reasonable request.
