# Supplementary material for: Lactate values during labour and their correlation with maternal and fetal outcome
Source: Reprod Fertil. 2026 Mar 24;7(1):RAF250140. doi: 10.1530/RAF-25-0140 (PMC13034522; doi:10.1530/RAF-25-0140)
Supplement: Supplementary file 1 [file supplementary_materials.pdf]

Supplementary Material

Figure 1: The flow diagram shows the enrolment process

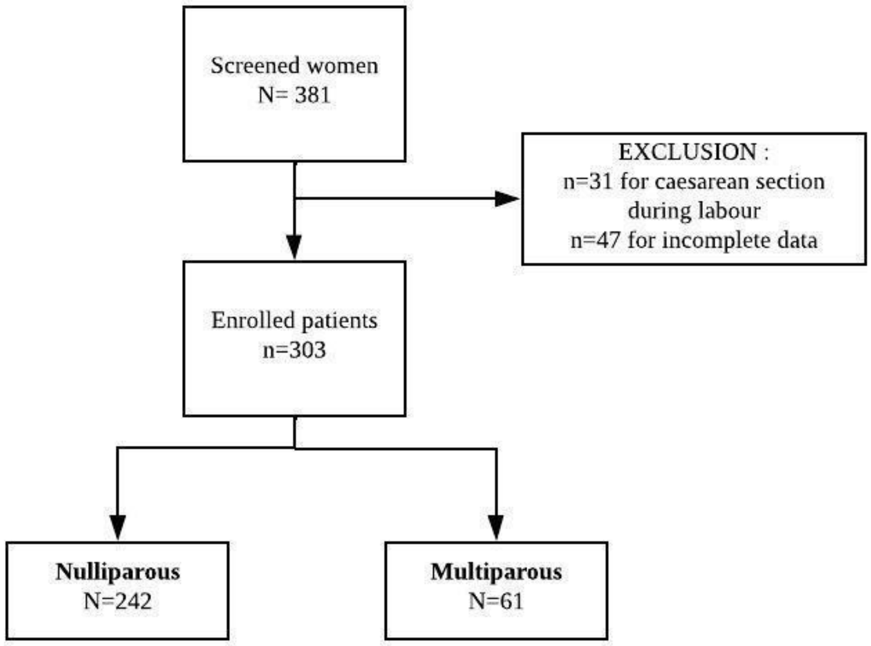

Table 1: Total number of patients divided by type of labour and blood loss

	Type of labour	Overall	< 500	≥ 500
**n**		**303**	**193**	**110**
**n (%)**	**1***	13 (4.3)	9 (4.7)	4 (3.6)
	**2°**	35 (11.6)	22 (11.4)	13 (11.8)
	**3^**	17 (5.6)	10 (5.2)	7 (6.4)
	**4§**	47 (15.5)	27 (14.0)	20 (18.2)
	**Spontaneous**	134 (44.2)	94 (48.7)	40 (36.4)
	**Spontaneous + Oxy**	57 (18.8)	31 (16.1)	26 (23.6)

Legend: 1*: Induction with only mechanical methods (double-balloon catheters or amniotomy); 2° induction with only prostaglandins (E2 vaginal or E1 oral); 3^ induction with only oxytocin; 4[§] induction with prostaglandins or mechanical methods and amniotomy and oxytocin.

Table 2: Lactate values at the three time points in different groups of induction of labour correlated with blood loss

	**Overall**	**< 500:1***	**> = 500:1***	**< 500:2°**	**> = 500:2°**
**n**	**112**	**9**	**4**	**22**	**13**
**LT0 (mean (SD))**	1.80 (0.67)	1.64 (0.57)	1.44 (0.20)	2.08 (0.72)	1.73 (0.58)
**LT1 (mean (SD))**	2.93 (1.49)	3.33 (2.16)	3.15 (1.22)	2.98 (1.44)	2.87 (1.97)
**LT2 (mean (SD))**	3.89 (1.59)	3.94 (1.65)	4.14 (1.29)	4.52 (1.98)	3.69 (1.66)
		**< 500:3^**	**> = 500:3^**	**< 500:4[§]**	**> = 500:4[§]**
**n**		**10**	**7**	**27**	**20**
**LT0 (mean (SD))**		1.83 (0.70)	1.72 (0.67)	1.90 (0.74)	1.55 (0.60)
**LT1 (mean (SD))**		2.78 (1.19)	2.92 (1.29)	3.09 (1.55)	2.53 (1.07)
**LT2 (mean (SD))**		3.48 (1.44)	3.29 (1.56)	3.72 (1.59)	3.89 (1.14)

Legend: 1*: Induction with only mechanical methods (double-balloon catheters or amniotomy); 2° induction with only prostaglandins (E2 vaginal or E1 oral); 3^ induction with only oxytocin; 4[§] induction with prostaglandins or mechanical methods and amniotomy and oxytocin.

Table 3: Lactate values at the three time points in spontaneous labour correlated with blood loss

	**Overall**	**<500:Spont**	**>=500:Spont**	**<500:Spont +Oxy**	**>=500:Spont +Oxy**
**n**	**191**	**94**	**40**	**31**	**26**
**LT0 (mean (SD))**	1.85 (0.69)	1.83 (0.56)	1.94 (0.90)	1.83 (0.76)	1.83 (0.70)
**LT1 (mean (SD))**	2.79 (1.22)	2.76 (1.30)	2.97 (1.06)	2.60 (1.24)	2.82 (1.15)
**LT2 (mean (SD))**	3.90 (1.55)	3.92 (1.62)	4.23 (1.61)	3.33 (1.20)	3.98 (1.48)

Figure 2 - Correlation of lactate at T0, T1 and T2 with blood loss

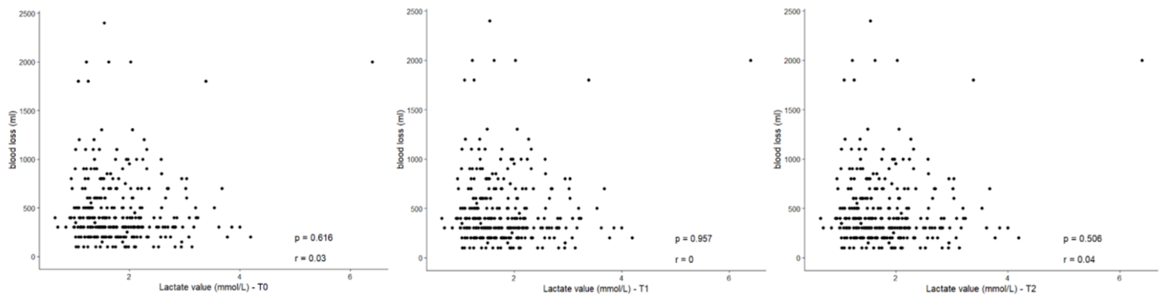

Figure 3: Contraction of the uterine smooth muscle

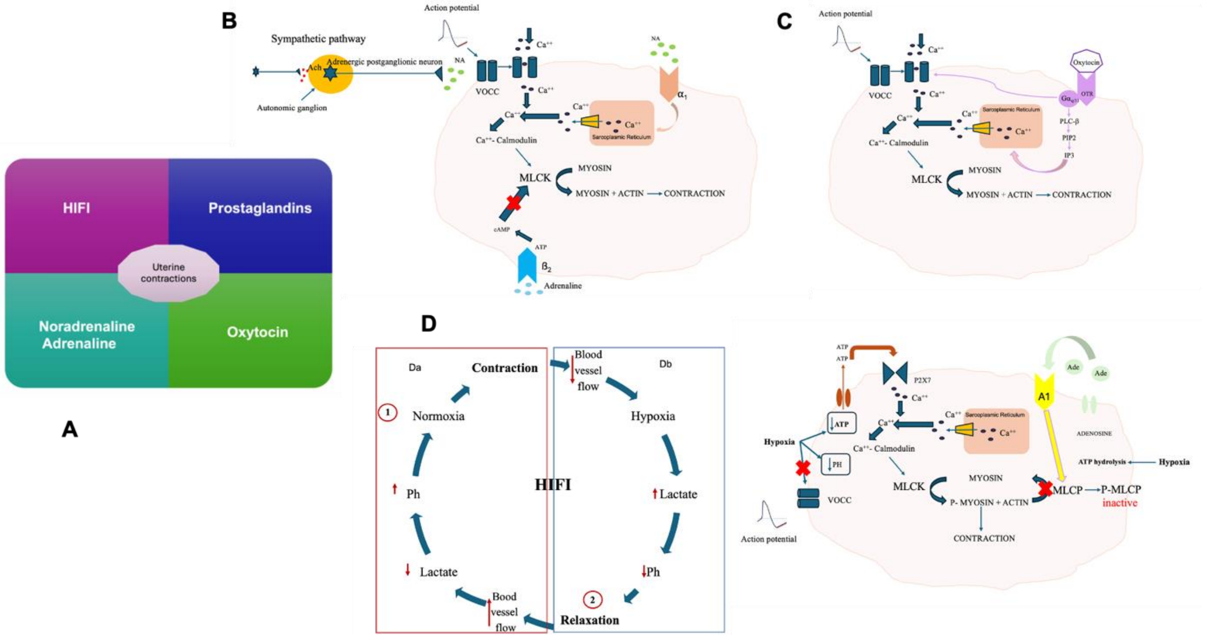

A. Rhythmic uterine contractions are controlled by several factors, including catecholamines, oxytocin, prostaglandins, and a complex process called hypoxia-induced force increase (HIFI), studied in pregnant rats’ myometrium.

B. Role of catecholamines

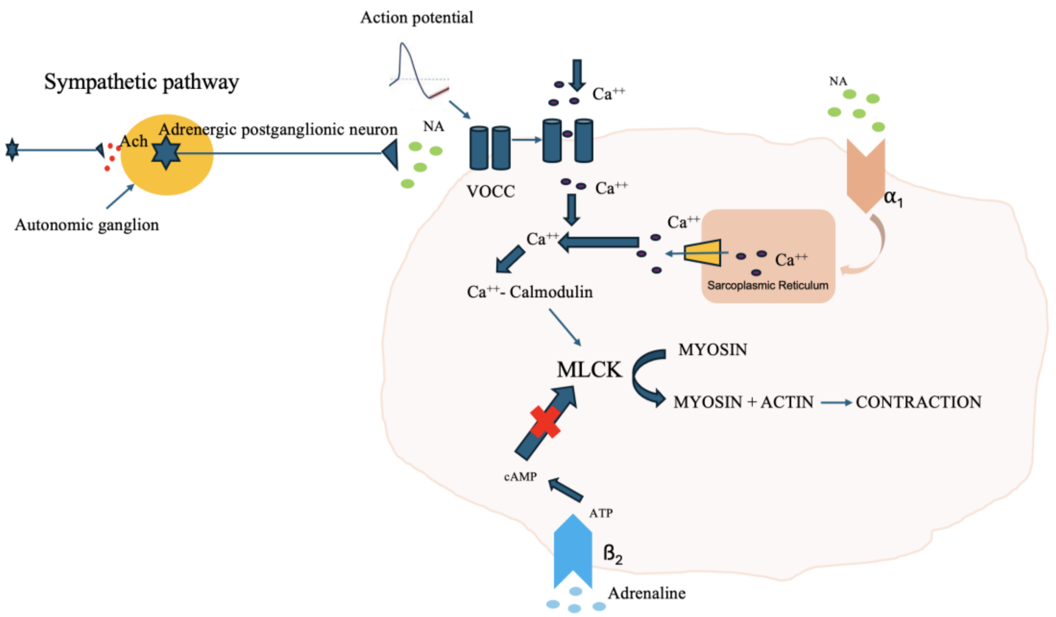

In the smooth muscle cell, the release of noradrenaline (NA) from the postsynaptic fibre activates voltage-operated calcium channels (VOCC), promoting calcium ions entry into the cell (Arrowsmith et al. 2014). The subsequent binding of calcium to calmodulin activates myosin light chain kinase (MLCK) by promoting the binding of actin and myosin (Arrowsmith et al. 2014). Noradrenaline, also produced by the adrenal medulla, binds to alpha-1 receptors, promoting calcium release from the sarcoplasmic reticulum and the binding of Ca-calmodulin (Hall and Hall 2021).
Additionally, adrenaline, also produced by the medulla, activates beta-2 receptors, increasing cyclic adenosine monophosphate (cAMP). This increase in cAMP activates protein kinase A (not shown in the figure) and causes the phosphorylation of MLCK by reducing the interaction between actin and myosin (Hall and Hall 2021).

C: Role of oxytocin

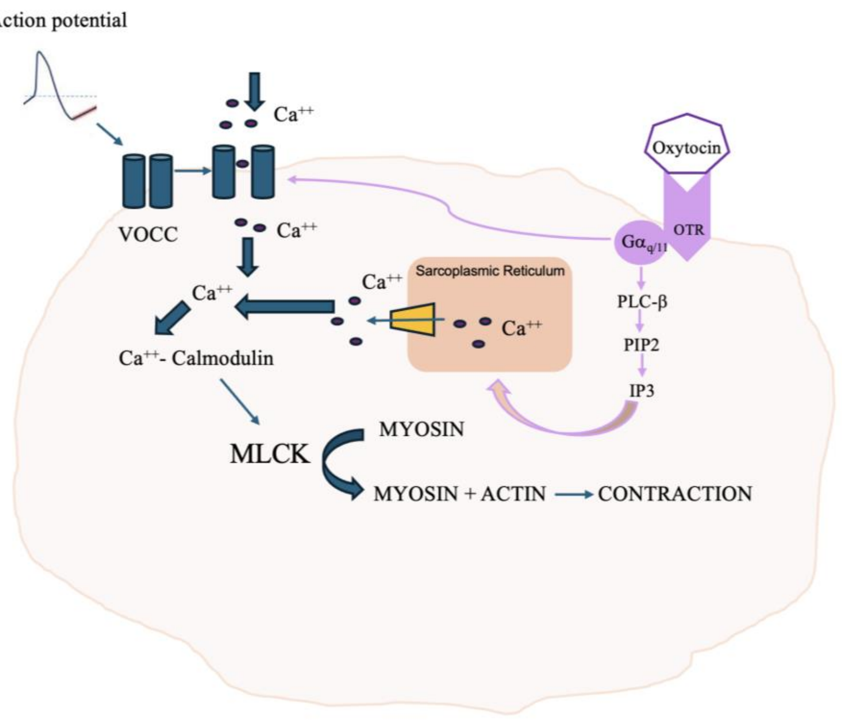

Oxytocin binds to its specific receptors (OTR), coupled to G protein. In the uterus, Gaq/11 activates phospholipase C-β (PLC-β), which hydrolyses phosphatidylinositol-bisphosphate (PIP2) to inositol-triphosphate (IP3) (Arrowsmith and Wray 2014). The release of calcium from the sarcoplasmic reticulum is promoted by IP3 so that calcium can bind to calmodulin (Arrowsmith et al. 2014). Calmodulin activates MLCK, promoting actin-myosin binding. In addition, the Gaq/11 protein

promotes the activation of VOCC. Furthermore, oxytocin has been observed to stimulate prostaglandin production, which sensitises the myometrium to oxytocin itself, increasing its receptors (Buckley 2015).

D: Role of HIFI

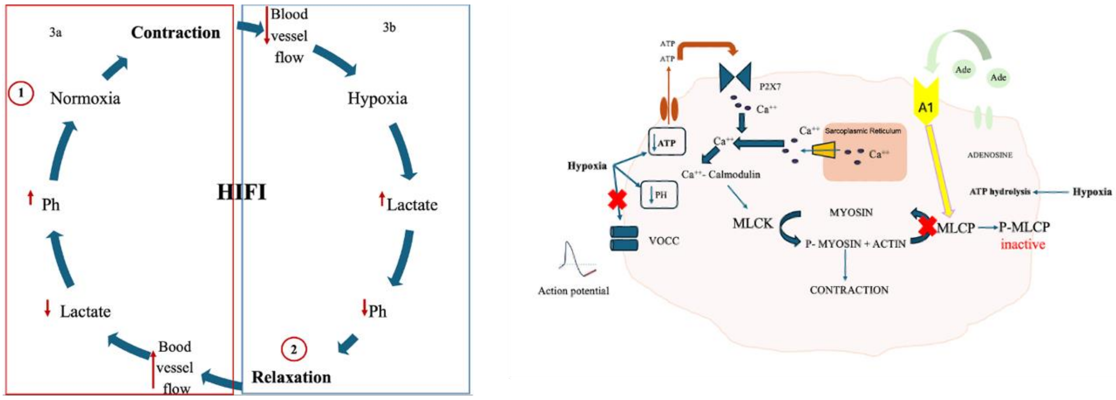

Legend: Da: normoxia phase   Db hypoxic phase

HIFI is a phenomenon in which the force of contraction in the uterus increases in response to a hypoxic state.

The myometrium contraction occludes uterine blood vessels and induces a brief hypoxic state, resulting in elevated lactate levels, a decreased pH and a reduced intracellular concentration of adenosine triphosphate (ATP), which promotes the subsequent relaxation of smooth muscle fibroblasts (Alotaibi et al. 2015).

Hypoxia inhibits VOCC and decreases myometrial contractility (Wray et al. 2021). However, the efflux of ATP from the myometrial cells promotes the activation of P2X7 purinergic receptors, preserving intracellular calcium concentration. Additionally, ATP hydrolysis has been shown to stimulate A1 receptors by increasing MLCK activity and decreasing the activity of myosin light chain phosphatase (MLCP). It is believed that these processes strengthen myometrial contractions (Alotaibi et al. 2015).
